# Effect of Inhibitor on Adsorption Behavior and Mechanism of Micro-Zone Corrosion on Carbon Steel

**DOI:** 10.3390/ma12121901

**Published:** 2019-06-13

**Authors:** Fengjuan Wang, Zhifeng Zhang, Shengping Wu, Jinyang Jiang, Hongyan Chu

**Affiliations:** 1 Jiangsu Key Laboratory of Construction Materials, School of Materials Science and Engineering, Southeast University, Nanjing 211189, China; fengjuan19921118@sina.com (F.W.); zzf_cool_0130@126.com (Z.Z.); shpwu1988@163.com (S.W.); 2 College of Civil Engineering, Nanjing Forestry University, Nanjing 210037, China; chuhongyan@njfu.edu.cn

**Keywords:** carbon steel, EIS test, XPS, interfaces, inhibition mechanism

## Abstract

A new type of inhibitor is studied in this paper. Inhibition efficiency and adsorption behavior of an inhibitor film on the steel surface is tested via the electrochemical method and theoretical calculation to establish the adsorption model. Test results confirm that inhibition efficiency is improved with the addition of an inhibitor, and the inhibitor film is formed firmly by comparing the characteristic peaks of S and N. Moreover, the micro-zone corrosion progress of Fe in 3.5% invasive NaCl-simulated seawater environment is studied. The results further show that corrosion is initiated under the zone without the inhibitor film, while it is prevented under the protection of the film. By the experiments, it is shown that inhibitor can be adsorbed on the surface of steel stably and has excellent protection performance for reinforced rebar, which can be widely used in concrete structure.

## 1. Introduction

Building materials comprise the most consumable materials in a modern city, especially concrete. However, concrete can be destroyed easily by static or dynamic loads, and concrete corrosion can take place anytime due to an adverse environment such as, fog and humidity, seawater, and alkaline or acidic soils [1]. Concrete structure destruction is mostly induced by the destruction of steel rebar in concrete. Generally, a passive film layer can be produced on the Fe surface in the environment of a high-alkalinity pore solution, and such a passive film can prevent the oxidation–reduction reaction on the steel surface [2]. However, a fragile passive thin film can be easily degraded by carbonization; CO_2_ transported to the surface of the steel bar reacts with Ca(OH)_2_ in the concrete, or chloride ions penetrate the surface of the steel causing corrosion to occur [3,4]. Therefore, to avoid the corrosion, many technological approaches have been developed to protect steel rebar, including coating with organic layers [5,6], polymer coatings [7], the formation of oxide layers [8], cathodic protection [9], and coating with other metals or alloys [10].

Nevertheless, due to the lower bond strength and short service-life durability incurred by using above methods, a new technology that has no impact on the bond strength between the steel and concrete structure and has a high service-life performance is required to ameliorate the current issues. In the past, inhibitors were used to prevent the permeation of detrimental ions from pore solutions into the surface of steel rebar, thus making the passive film immune to erosion by carbonation and chloride ions. Zarrouk et al. [11] studied the relevant inhibition properties, especially adsorption behavior, and theoretically calculated the effect of inhibitors in a hydrochloric acid solution on carbon steel. Studies show that the inhibition efficiency has a positive correlation with the inhibitor concentration, but a negative correlation with the temperature. Zhang et al. [12] researched oxo-triazole derivatives used as a corrosion inhibitor for mild steel in an acidic solution. Liu et al. [13] researched ginger extract as a green inhibitor for carbon steel in simulated concrete pore solutions. Verbruggen et al. [14] researched inhibitor evaluation in different simulated concrete pore solution for the protection of steel rebars. Neither the results of weight-loss measurements, or of electrochemical tests using such materials have shown satisfactory protection of mild steel against corrosion.

Beyond the above-mentioned studies, we used the scanning vibrating electrode technique (SVET) to study the anticorrosion mechanism under an invasive environment with and without the protection of a new inhibitor, bismuth-thiol, and visually display the corrosion process.

## 2. Experimental Methods

### 2.1. Materials and Sample Preparation

**Figure 1:**
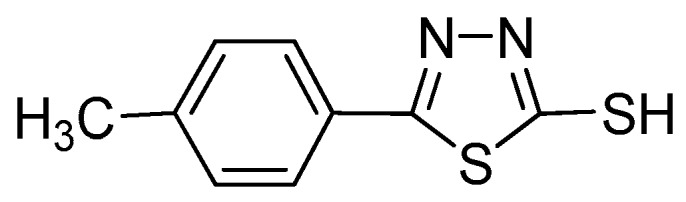
Structure of inhibitor molecule.

The molecule of the inhibitor under study, namely 2-(5-mercapto-1,3,thiadizaole-2-yl)-(4-methylbenzene), is shown in Figure 1. In the concentration studies, the concentration of the inhibitor in a simulated concrete solution (SCP) with 3.5% NaCl ranged from 0.1 to 5.0 mmol/L with a natural pH at an ambient temperature. The simulated concrete solution is a mixture of KOH (28.0 g/L), NaOH (8.0 g/L), and Ca(OH)_2_ (2.2 g/L), and the pH of the simulated concrete solution is 13.6. This solution in the absence of the inhibitor is taken as the blank for comparison.

Commercial Q235 steel rebar (10 × 10 × 10 mm^3^, which contains 0.14–0.22 C, 0.30–0.65 Mn, <0.60 Si, <0.05 S, <0.045 P (wt%) and Fe balance) is used in this study. Before the experiment, the rebar specimens are polished, degreased ultrasonically, and then dried using the methods described in References [15,16]. All samples tested are immersed in a prepared solution (SCP with 3.5% NaCl) at ambient temperature and exposed to air.

### 2.2. EIS Measurements

Electrochemical impedance spectrum (EIS) experiments are conducted on a potentiostat/galvanostat/zero-resistance ammeter (Gamry Instruments 1000, Gamry Instruments Corp., USA) using the usual three-electrode test setup. The ac frequency ranged from 1 × 10^5^ Hz to 1 × 10^−2^ Hz.

### 2.3. Surface Chemical Composition Measurements

Before conducting X-ray photoelectron spectroscopy (XPS) measurements, as-prepared cubic steel (4 × 4 × 2 mm^3^) is soaked for 3 days in SCP with NaCl and inhibitor with a concentration of 3.5 wt% and 5.0 mmol/L to form the firm inhibitor film. Under the same conditions, carbon steel soaked in a solution without the inhibitor is prepared for comparison.

The XPS measurements of the surface chemical composition are carried out on a scanning microprobe (PHI Quantum 2000, Physical Electronics, Inc., Chanhassen, MN) with an Al *K*α radiation source. Parameter settings and test procedures are based on Reference [17].

### 2.4. SVET Measurements

The SVET measurements of the carbon steel (4 × 4 × 2 mm^3^) are conducted using the same process as the XPS measurements. Before the SVET tests, however, scratches are made on the surface of the carbon steel. The tests are then conducted in the invasive environment of a pure 3.5% NaCl solution.

Scanning electrochemical workstation (Applicable Electronics, Inc., Chanhassen, MN) is used to conduct the SVET measurements. Test setup is based on Reference [18].

## 3. Results and Discussion

### 3.1. EIS Measurement Results

**Figure 2:**
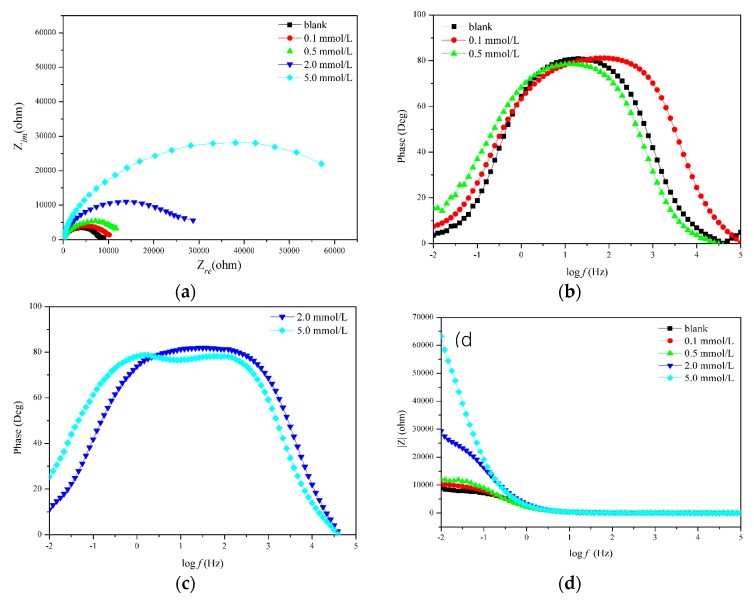

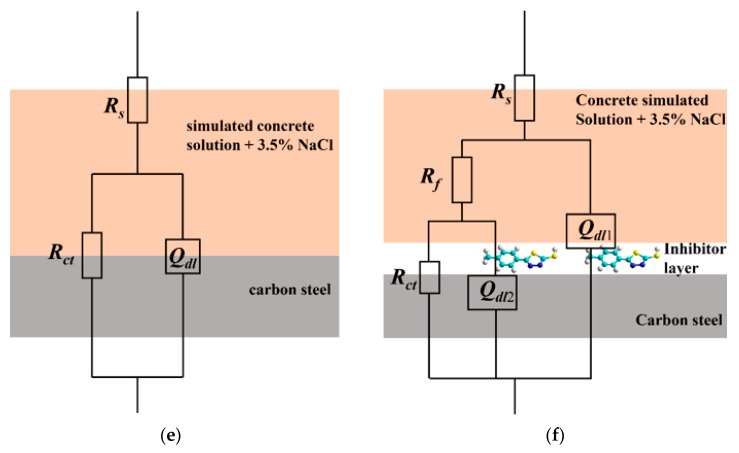
EIS spectra of Q235 carbon steel in simulated concrete solution with 3.5% NaCl in the absence of and with varied inhibitor concentrations: (**a**) Nyquist plots; Bode plots of (**b**) blank sample, 0.1 and 0.5 mmol/L, (**c**) of 2.0 and 5.0 mmol/L, and (**d**) of |*Z*|; equivalent circuit with (**e**) one-time constant and (**f**) two-time constants.

A set of Nyquist and Bode plots of carbon steel in as-prepared solutions with varied concentrations of the inhibitor is shown in Figure 2a, which visually displays the capacitive and resistive behavior at the interface between the solution and the adsorbed inhibitor layer [15].

As is known, charge transfer resistance *R*_ct_ represents the developing tendency of capacitive loop, and double layer capacitance of *C*_dl_ represents the coverage percentage of the inhibitor on the carbon steel surface. A lower value of *C*_dl_ indicates a high coverage of inhibitor on the surface, which can provide a better anticorrosion/corrosion-protection effect [17]. In Figure 2a, compared to the bare sample, the larger diameter of the capacitive loop and the increase of the capacitive loop with increasing concentration both show that the inhibitor provides stronger corrosion protection for Q235 carbon steel.

Bode plots of the inhibitor at varied concentrations are shown in Figure 2b–d. From the Bode plots of the blank and inhibitor concentrations of 0.1 and 0.5 mmol/L, there is one time constant, which is probably associated with the charge-transfer process [19]. On the contrary, there are obviously two time constants for the sample with concentrations of 2.0 and 5.0 mmol/L.

In order to analyze the impedance characteristics, an equivalent circuit is proposed to fit the impedance spectra, as shown in Figure 2e,f. In the figure, plots with one time constant are fitted by the circuit shown in Figure 2e, while the balance is fitted by the circuit shown in Figure 2f.

The impedance values of the equivalent circuit and electrochemical parameters are given in Table 1. When the ***R*_ct_** values are obtained, *IE*% values of the inhibitor are calculated by [15]:

(1) $$\mathrm{IE}\%\;=\;(R_{\mathrm{ct}}-R_{\mathrm{ct}0})/R_{\mathrm{ct}}\times 100$$

where, ***R*_ct_** and ***R*_ct0_** are the respective charge-transfer resistance values of the previously described as-prepared solutions containing the inhibitor and inhibitor-free. By comparing the ***R*_ct_** values, it is clear that the more inhibitor molecules there are, the better the inhibition efficiency.

Owing to the fact that corrosion could not occur at the covered sites, the molecules of inhibitors adsorbed on the Fe surface can supply a barrier that prevents the corrosion process. Therefore, the more barriers that are on the Fe surface covered by the inhibitor molecule film, the higher the inhibition efficiency regarding the corrosion process [17].

### 3.2. Adsorption Isotherm Analysis

**Figure 3:**
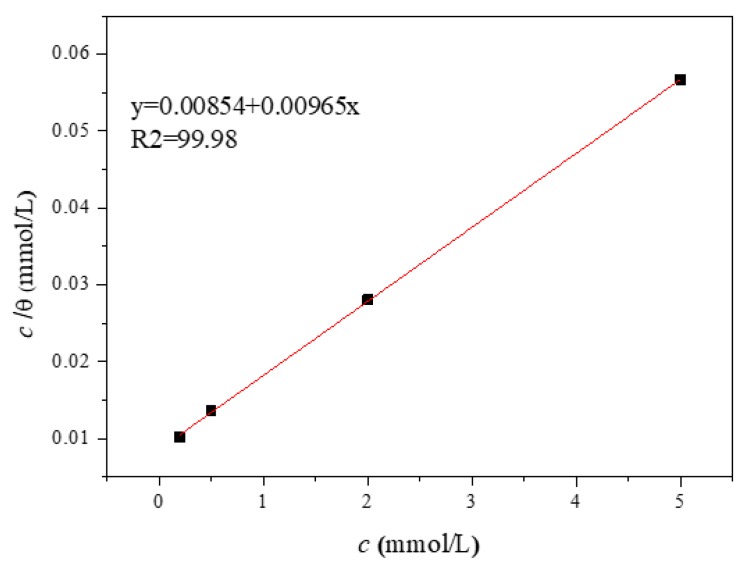
Adsorption isotherm of inhibitor on Q235 carbon steel surface in simulated concrete solution with 3.5% NaCl and different inhibitor concentrations.

Adsorption isotherm is usually used to build the interaction mode between the inhibitors and the Fe surface [20]. Langmuir adsorption isotherm is cited based on the EIS test results to study the adsorption behavior of the inhibitor on Fe surface. The following formula can be used to express the Langmuir adsorption isotherm [21]:

*c*/θ = 1/*K*_ads_ + *c* (2)

in which, *c*, *θ*, and *K*_ads_ represent the concentration of inhibitor (mmol/L), coverage equivalent (defined as *IE*%), and equilibrium constant of inhibitor adsorption (L/mol), respectively. *c*/θ versus *c* is plotted in Figure 3 which is a straight line and the slope is close to 1, showing that Langmuir adsorption isotherm is the fittest to describe the behavior of inhibitor on the Fe surface [22].

By the calculation of *K*_ads_ and the following equation [23], the standard adsorption free energy (Δ*G*^0^_ads_) can also be obtained:

(3) $$\Delta G_{\mathrm{ads}}^{0}\;=\;-\mathrm{RT}\ln(55.5K_{\mathrm{ads}})$$

where, *R*, *T*, and the value 55.5 are the universal gas constant (J·mol/K), temperature (K), and molar concentration (mol/L) of water in solution, respectively.

In this paper, due to the calculated value of Δ*G*^0^_ads_ is −21.25 kJ/mol, we can know that it is spontaneous for the adsorption behavior of inhibitor molecule, which contains physisorption and chemisorption together, caused by electrostatic interactions and covalent bonds, respectively [24].

### 3.3. XPS Analysis

**Figure 4:**
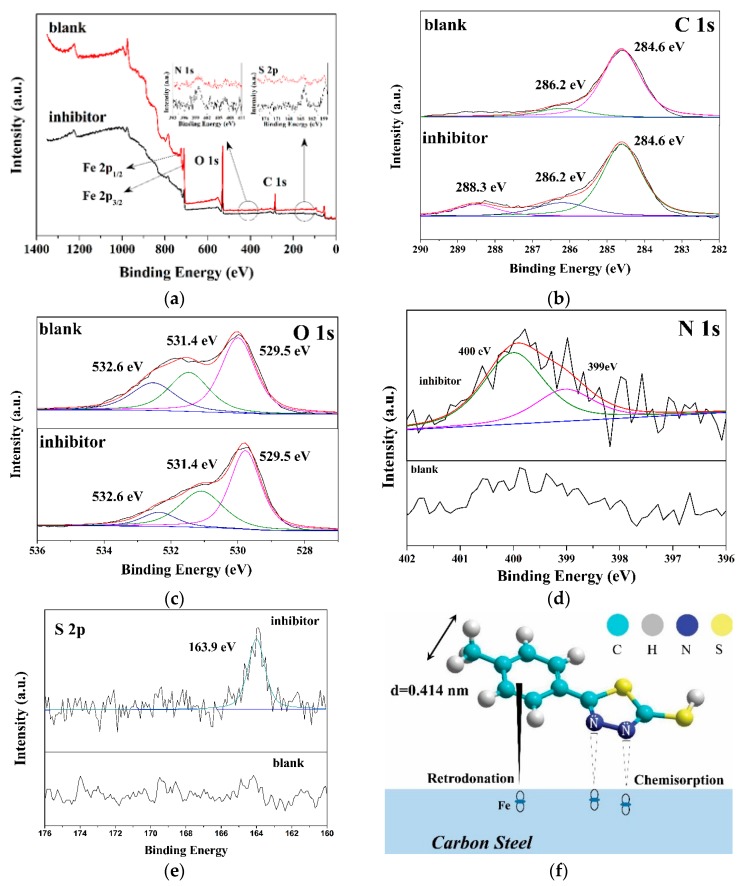
XPS spectra of (**a**) total spectra; high-resolution spectra of (**b**) C 1s, (**c**) O 1s, (**d**) N 1s, and (**e**) S 2p peaks; (**f**) schematic of inhibitor adsorbed on carbon steel surface.

XPS measurement is a useful tool to identify the combination mode between elements and the substrate. Therefore, the inhibitor adsorbed on the carbon steel surface is measured using XPS to prove that the inhibitor is adsorbed on the surface and to explain the combination process. Figure 4 shows total spectra (Figure 4a), as well as high-resolution C 1s (Figure 4b), O 1s (Figure 4c), N 1s (Figure 4d), and S 2p (Figure 4e) XPS spectra, for samples with inhibitor and inhibitor-free, no other impurity ions are detected. In the C 1s, O 1s, N 1s, and S 2p regions, the deconvolution of multiple peaks is performed to determine the respective binding energies [25].

As shown in Figure 4b,c, it can be seen that there is no obvious difference between the samples with an inhibitor and the blank sample for the high-resolution of O 1s XPS spectra, especially the peak at 288.3 eV is attributed to the sp^2^-hybridized carbon [26,27], which we can speculate comes from the inhibitor molecule.

As shown in Figure 4b, two characteristic peaks of 284.6 eV and 286.2 eV are observed in the inhibitor and blank samples, while the binding energy of 288.3 eV is only detected in the sample with inhibitor. The peaks at 284.6 eV and 286.2 eV are ascribed to a C–C bond and C=O bond, respectively, which is attributed to the adventitious hydrocarbon from the XPS instrument itself [28]. The peak at 288.3 eV is attributed to the sp^2^-hybridized carbon [26,27], which we can speculate comes from the inhibitor molecule. Figure 4c shows the high-resolution of O 1s XPS spectra, the first component at a binding energy of 529.5 eV is assigned to the Fe oxide, such as FeO and Fe_2_O_3_ [29,30]. The second peak, with a binding energy of 531.9 eV, can be considered to result from the hydroxide bonds chemisorbed on the surface [31,32]. The last peak, at 533.6 eV, can be attributed to O of the adsorbed water [33]. From Figure 4c, it can be seen that there is no obvious difference between the samples with an inhibitor and the blank sample for the high-resolution of O 1s XPS spectra.

In Figure 4d, a broad N 1s peak in region of 396–402 eV with a maximum located at a binding energy of 399.7 eV is clearly identified for the inhibitor sample. Two Gaussian–Lorentzian peaks of the high-resolution N 1s spectrum exist: One at a binding energy of 399.0 eV that could be attributed to the type of C–N bond [34], and the other at 400 eV that could be assigned to the bond of N adsorbed on the carbon steel surface [35]. In comparison, no obvious N 1s peak is detected in the blank sample. Based on the results of the analysis of the high-resolution N 1s XPS spectrum, we can conclude that it is the formation of chemical bonds between elemental N and Fe that facilitate the adsorption of the inhibitor on the carbon steel surface for this type of inhibitor, which is consistent with the findings of Reference [36]. In Figure 4e, it can be seen that there is a peak located at 163.9 eV, which corresponds to the C–S bond [37]. In contrast, no S 2p peak can be seen in the spectrum of the blank sample.

Therefore, by integrating the results of the adsorption isotherm and XPS analysis, we can further surmise that physisorption and chemisorption coexist, as a result of electrostatic interactions for the former and of N–Fe chemical bonds for the latter. Based on the above analysis, the adsorption mechanism of the inhibitor with coexisting physisorption and chemisorption on carbon steel is depicted in Figure 4f.

### 3.4. SVET Analysis

**Figure 5:**
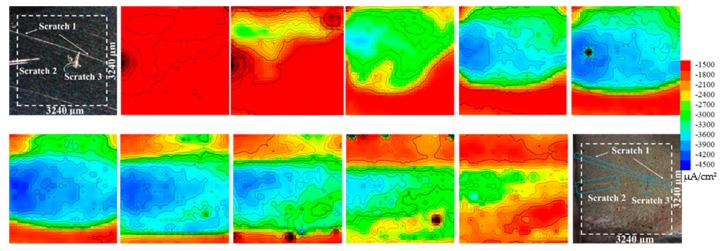
Corrosion progress of sample in NaCl solution measured by scanning vibrating electrode technique (SVET) test.

SVET is used to measure the potential differences on the Fe surface in a solution with an extremely high resolution ratio due to the fluxes of ionic current, which is caused by the electrochemical reactions occurring at the Fe surface [38]. Distribution of the local current density over a surface can be depicted in SVET as two-(2D) or three-dimensional (3D) current–density maps [39]. Figure 5 depicts the SVET 2D maps obtained from carbon steel samples, each immersed in 3.5% NaCl for 15 min. The first and last photographs in the figure are of the samples before and after the immersion test, in which the valid test area measures 3240 × 3240 μm^2^, on the surface of which three scratches are made to distinguish the blank steel sample from the steel sample that has adsorbed the inhibitor. The three scratches are designated as Scratch 1, Scratch 2, and Scratch 3.

In the first 15 min, the sample presents a uniform corrosion feature and the current density is nearly −1500 μA/cm^2^. Notably, the current density at the Scratch 2 location is slightly higher than in any other location. As the corrosion process continues, the current density continuously increases, and corrosion occurs at the locations of all three scratches. After the SVET test, the scratch locations cannot be distinguished clearly. The maximum current density is −4500 μA/cm^2^, which is three times higher than that recorded in the first 15 min. After immersion in 3.5% NaCl for 2 h, the size of the corrosion-zone area begins to decrease due to the coverage of the corrosion product, which blocks the probe from detecting the current variation. The current density remains invariant in the entire immersion period at the localized zone of the inhibitor film, indicating that the inhibitor film provides the steady protection from corrosion for the carbon steel.

Two matters must be discussed. First, while the scratches are small, the corrosion zone is large. This may have been caused by the fact that, when the scratches are carved on the surface of the carbon steel, the inhibitor film is wiped off along with the removal or loosening of the adjacent film, since the inhibitor film is connected to the adjacent film in the close-packed structure. Second, invasive Cl^−^ ions cannot break the inhibitor film directly. The process of Cl^−^ ions invading the carbon steel in the zone of the inhibitor film is, (1) it destroys the carbon steel in the transition zone between the inhibitor film and the blank zone, and (2) then strips the inhibitor film bit by bit until the inhibitor film is completely destroyed. Using 2D maps of the SVET test results, the corrosion process under the protection of an inhibitor is vividly illustrated.

## 4. Conclusions

The results obtained in this work show that it is effective for an inhibitor of bismuth-thiol to prevent the corrosion process of the carbon steel in the simulated concrete pore solution with erosive NaCl, and that the inhibition efficiency increases with increasing inhibitor concentration. Subsequently, the existence of adsorption layers formed by the inhibitor on the carbon steel is confirmed by the XPS analysis through detecting the characteristic atoms of S and N. The corrosion mechanism and process are also verified by SVET measurements, which show that under the protection of inhibitor the current density of the inhibitor zone is much lower than that of the blank zone. Considering the above results, we determine that it is the inhibitor film that plays the vital role in resisting the corrosion process and, in particular, that the progress of corrosion can be stopped under the protection of an intact protective inhibitor film.

As the rust inhibitor is a kind of small organic molecule that can be present in the pore solution of concrete, it has no effect on the long-term service performance of the concrete. This thesis shows that this rust inhibitor has perfect rust resistance performance for steel bars. In the subsequent researches, it is necessary to develop or select a migration rust inhibitor with similar molecular structure in order to study the repair effect that the rust inhibitor migrates to the surface of the steel through the concrete to repair the damaged passivation film on the steel bars when the concrete structure begins to break.

## Figures and Tables

**Table 1:** EIS parameters for corrosion of Q235 carbon steel in simulated concrete solution (3.5% NaCl) without and with different inhibitor concentrations.

Sample (mmol/L)	*R_s_* (Ω cm^2^)	*Q_dl_*_1_ (F cm^−2^)	*n*1	*R_f_* (Ω cm^2^)	*Q_dl_*_2_ (F cm^−2^)	*n*2	*R_ct_* (Ω cm^2^)	*η* (%)
Blank	4.382	6.89 × 10^−5^	0.9245	–	–	–	7783	–
0.1	1.191	7.316 × 10^−5^	0.9004	–	–	–	9691	19.7
0.5	6.545	8.83 × 10^−5^	0.8892	–	–	–	12,320	36.8
2.0	1.543	4.831 × 10^−5^	0.9374	274	1.890 × 10^−5^	0.4388	32,060	75.7
5.0	2.679	4.539 × 10^−5^	0.9375	678.4	2.665 × 10^−5^	0.7467	70,650	89.0
